# Suicide Prevention: University Students’ Narratives on Their Reasons for Living and for Dying

**DOI:** 10.3390/ijerph18158029

**Published:** 2021-07-29

**Authors:** Ines Testoni, Silvia Piol, Diego De Leo

**Affiliations:** 1 Department of Philosophy, Sociology, Pedagogy and Applied Psychology (FISPPA), University of Padova, 35131 Padova, Italy; silvia.piol.1@studenti.unipd.it; 2 Emili Sagol Creative Arts Therapies Research Center, University of Haifa, Haifa 3498838, Israel; 3 Australian Institute for Suicide Research and Prevention, Griffith University, Brisbane 4122, Australia; d.deleo@griffith.edu.au

**Keywords:** suicide, suicide prevention, Death Education, social work, social support, college students

## Abstract

Social isolation and loneliness are increasing in our contemporary western society and seem to correlate with suicide in adolescents and young adults. Social Workers are a potential resource to create such initiatives and projects that promote inclusion and cohesion within communities, a protective factor against suicide. Sixty-two Social Work BA students participated in a Death Education course based on education on suicide prevention. Participants carried out two activities. First, they were invited to complete two written semi-structured interviews on young people’s reasons for living and dying. Second, they were invited to design suicide prevention interventions targeted at their peers and adolescents. Data were analyzed qualitatively within the Thematic Analysis framework. As regards the first activity, four main themes were identified: (1) Internet and social media; (2) social isolation and loneliness; (3) the importance of proximal relationships; and, (4) the importance of networking between proximal relationships, educational institutions and mental health services. Whereas, as for suicide prevention interventions, three main ideas were identified: (1) suicide prevention through community and networking between services; (2) academic institutions: high schools and universities; and, (3) suicide prevention through new technologies. To conclude, Death Education as education on suicide prevention can offer young people a space in which to voice their and their peers’ reasons for living and dying and to reflect upon their contribution to suicide prevention as students and as future professionals.

## 1. Introduction

### 1.1. Death Education as Education on Suicide Prevention

The World Health Organization (WHO) [1] estimated that in the year 2016 almost 800,000 people took their lives in the world, that is a global age-standardized suicide rate of 10.5 per 100,000. The WHO declared suicide a health priority [2]. With reference to young people, a recent meta-analysis revealed that the aggregate lifetime and 12-month prevalence of suicide attempts is 6% and 4.5%, respectively; for suicidal plan it is 9.9% and 7.5%, respectively; and for suicidal ideation it is 18% and 14.2%, respectively [3]. Moreover, suicide is the second leading cause of death among those aged between 15 and 29 worldwide, for both sexes [1]. Italy shows a similar situation: suicide is accounted as the third leading cause of death—after road injuries and cancer—among young people aged 15–24 [4]. Finally, given that suicides have a great impact in terms of years lost due to disabilities (YLDs) and every juvenile suicide represents as many as 60 potential years of life lost (YLLs) [5], suicide among adolescents and young adults should be regarded as a major health concern, and appropriate ad hoc prevention interventions should be realized.

Given the time spent by young people in formal schooling, it would be important to include suicide prevention education within teaching curricula [6,7]. This type of intervention falls under the category of universal prevention, defined by Mrazek and Haggerty [8] as a form of prevention targeted at either the general population or a group within it. Universal school suicide prevention interventions are aimed at raising awareness among students on warning signs and risk factors for suicide in young people and at promoting a positive attitude toward help-seeking for at risk peers [6,9]. Notwithstanding resistance from teachers toward such interventions (as they are concerned they might foster suicidal thoughts in young people), some studies show that there would be no iatrogenic effects [6,10]. If anything, such interventions would increase students’ ability to identify at risk peers, be more prone to seek help for them and would positively impact on suicide-related outcomes. Moreover, stigma reduction toward mental health problems and suicidality fostered through suicide prevention education can increase people’s intention to seek help in times of crisis [11,12]. 

Death Education (DeEd: [13]) can be employed as a type of education about suicide prevention for young people in formal schooling [7]. DeEd creates a safe space for reflection on existential themes, thus offering an opportunity to talk about death and the human condition as mortals and finite beings. The need for such spaces stems from the changes brought about by the postmodern era, such as a great increase in well-being and the secularization of society with the consequent removal of any form of reflection upon death and human limits [14]. The awareness of finitude, in turn, makes humans appreciate their time spent on Earth, thus valorizing life and strengthening their will to safeguard it [7,15]. DeEd is aimed at creating an appropriate language around death and death-related issues, creating a space to recognize, experience and share emotions and feelings derived by thinking about death, reasoning on human limits and finitude and on the meaning of life, to reduce death anxiety and create a safe space for students to share their learning experiences, together with their thoughts and feelings [16]. 

### 1.2. Community Social Support as a Promising Protective Factor for Suicide 

When moving from individual to social risk factors for suicide, researchers suggest that chronic loneliness could be a possible risk factor for suicidal thoughts and behaviors (STBs), probably due to its association with depressive symptomatology [17,18]. Research suggests that there is a correlation between loneliness and depression, loneliness and STBs, and between the lack of social connection and suicide [19,20,21]. Loneliness—defined as a discrepancy between an individual’s preferred and actual social relations [22]—seems to be highly prevalent in the Z generation and among young adults [23,24], although the literature concerning loneliness in young people is still scant. The literature [25] suggests that this might be due to risk factors inherent to the developmental phase, such as moving out from their parents’ houses and their communities, and having to create new social networks. In accordance with the Interpersonal Theory of Suicide [26], loneliness, social isolation and thwarted belongingness can predict suicidal thoughts and behaviors [27] and correlate with STBs in university students [19,28,29]. 

This phenomenon might be understood as a byproduct of our contemporary western society, which is becoming more and more individualistic, thus being unable to create opportunities for social aggregation within communities, as indicated by the social capital framework [30]. Social capital—which has been defined as “social networks and the norms of reciprocity and trustworthiness that arise from them” [30] (p.19)—originates from the work of the sociologist Emil Durkheim, who posited the existence of an inverse relation between social integration and the number of suicides within a specific society [31]. This line of reasoning constitutes a possible explanation for the regional distribution of suicides in Italy, showing much lower suicide rates in the South and the Isles compared to the rest of Italy [32,33]. Ghirini and Vichi [32] suggest that, although they have not yet been explored, sociocultural factors, such as family structure and relationships within and beyond the family, might be responsible for this distribution.

The literature finds positive correlations between dense and strong horizontal networks, meaning social ties with family, relatives, friends and colleagues, and mental health outcomes [31]. The literature also shows promising insights for the role of community social support as a potential protective factor for suicide [33,34]. It therefore seems useful to strengthen the social dynamics that build and reinforce community ties, in both concrete and virtual social reality.

### 1.3. Aims

Given the potential of community social support in suicide prevention, this research is aimed at understanding future social workers’ perspectives on their potential as professionals in suicide prevention. The aim of the project was to create a Death Education opportunity for Social Work university students. This was meant to foster a discussion on the potential for social workers in creating initiatives and projects promoting inclusion and cohesion within communities as a protective factor against suicide in adolescents and young adults.

The study sought to answer the following research questions: “How do Social Work university students understand suicidal choices by adolescents and young adults?”, “Which are—according to their perspective—the main reasons driving young people to either choose to live or die?” and “How can Social Work university students employ their creativity, age and professional skills to create suicide prevention interventions to prevent suicide in adolescents and young adults?”. 

## 2. Materials and Methods

### 2.1. Study Design and Sampling

Participants were third year Social Work Bachelor’s Degree students from a university in northern Italy, attending a course held by the first author. The recruitment period was in the fall semester of the year 2019. Out of the 110 college students attending the latter course, 62 decided to participate in the present research, that is 57 females (91.9%) and 5 males (8.1%). Their age spanned between 20 and 26 years (mean age = 21.6 and standard deviation = 1.12). Almost all participants were unmarried (98.4%). No information was gathered on possible past personal experiences of suicide attempts by participants. The aforementioned course was a required one pertaining to the field of social psychology and explored the importance of active citizenship, empowerment and collective action in order to promote health in the community. The course was organized in the form of a Death Education intervention, fostering reflection around different facets of death and dying, with the aim of re-creating a language around these topics and of raising awareness on one’s mortality. During the course, the debated death-related topics spanned from palliative care, end of life, grief and suicide, with a greater focus on the latter and, more specifically, on suicide and suicide prevention in adolescents and young adults. All these topics were explored in relation to the role of social workers.

The present research was organized around two main activities. The first activity regarded students’ interviews. Following the experience of Marsha Linehan [35], students were first guided by the professor in charge to reflect and brainstorm upon adolescents and young adults’ reasons for living and dying; then, participating students were invited to complete two written semi-structured interviews on young people’s reasons for living and dying [6]. Marsha Linehan specifically adopted a perspective according to which it is possible to address the issue of suicide as a problem that affects everyone and that can be addressed and discussed to create a shared awareness of the problem. Communication is, according to the author, the first step to activate the dialectic between acceptance and change. Being able to talk about it in university courses allows for a breakdown of the prejudice that causes the topic to be censored [35]. Therefore, the course began by discussing with the students about why they think people they know or themselves did not carry out the act when faced with suicidal ideation. The discussion aimed to make students understand and recognize their reasons for living. The goal was then to think of projects that would reinforce those reasons. The second activity regarded project works, that is the creation of suicide prevention interventions. In line with the principles of student-participatory learning successfully applied to Death Education courses [36], students were invited to choose their group members and split into 10 groups of between 6 to 8 students. Each group had to create a suicide prevention intervention and, according to their interests and knowledge on suicide risk factors, choose a target for their interventions in suicide prevention. The general idea of the entire research/intervention project is that social workers can act in communities to activate support groups for adolescents and young adults, in order to prevent suicide. Therefore, in this course we wanted to strengthen this type of expertise and future professional awareness.

The research followed the APA Ethical Principles of Psychologists and Code of Conduct and the principles of the Declaration of Helsinki, so participants were explained in detail all the objectives of the research and the methodology of analysis used. They were asked permission to record the conversations, to transcribe their answers and to analyze their contents in order to study the phenomenon. Participation in the research was made sure to be voluntary. Participants were guaranteed that the content of their interviews would remain confidential and only those who had given written signed consent participated in the research. All the names cited below are fictitious (pseudonyms) and the quotations have been slightly changed to prevent any possibility that participants could be identified. The study was approved by the Ethics Committee for Experimentation of the University of Padua (n. 83C0520DD0185D56F9799AA5E302EDA9).

### 2.2. Data Collection

As regards the first activity, the study employed a semi-structured interview format. Students who expressed their wish to participate in the research were invited by the first and second author to respond to two written interviews. The two interviews were carried out as one close to the beginning of the course and the second one close to the end of the course. In the first interview, participants were invited to reflect upon and share their ideas about young people’s reasons for dying, whereas the second interview was about young people’s reasons for living. More specifically, in the first interview participants were invited to respond to the question: “Why is life refusal considered a solution by some young people?”; whereas, in the second interview, they responded to the question “Which are the reasons driving young people to choose life?”. Overall, 124 interviews were obtained, 62 on reasons for dying and 62 on reasons for living. Participants were assigned one week for each interview and no space limit was given. As regards the second activity, students worked on designing suicide prevention interventions for young people, adolescents and young adults. They worked on their projects the whole semester and presented and shared their projects and their photovoice activities with their classmates at the end of the course. In designing the interventions, students had to first focus on the problem analysis and the preferred specific target for their intervention among young people. They then had to think about the methodology they wished to use to prevent suicide within their target group. Moreover, they had to hypothesize the territory and setting of the intervention, the team delivering the intervention and the expected costs. Finally, students had to consider effectiveness measurements and dissemination opportunities. Moreover, given the importance of the arts and creativity in Death Education interventions [37], participants were also invited to capture the meaning of their projects through photography. More specifically, they engaged in a photovoice activity, that is the employment of photography to define a problem within a community and then elaborate possible solutions to it [38]. Students had to consider a possible future photographic exposition with the aim of raising awareness on the specific topic or to fundraise to bring about new solutions to the problem. With the photographic exposition in mind, each student took one photograph expressing their understanding of the problem or of potential solutions, and added a short caption. Finally, students had to present their photographs and captions to their colleagues at the end of the course. 

### 2.3. Data Analysis

Qualitative data analysis was adopted in this study, which aims to understand a phenomenon by exploring the perspective of those directly experiencing it [39]. The authors chose to employ Thematic Analysis as a methodology for data analysis [40]. This methodology allows to identify, analyze and finally report patterns or themes inside the data. The data analysis process attempted to follow the stages identified by the literature. Firstly, researchers read the interviews in order to familiarize themselves with the data. Secondly, they read the interviews a second time and identified significant portions of text, called quotations, to which codes were assigned. Thirdly, they performed a second analysis and tried to identify themes within the data. Finally, a narrative was found among the themes and the results were compared with existing literature. The data were analyzed using Atlas.ti 8th version (ATLAS.ti Scientific Software Development GmbH, Berlin, Germany).

## 3. Results

### 3.1. Adolescents’ and Young Adults’ Reasons for Living and Dying

The analysis identified four main themes: (1) Internet and social media; (2) social isolation and loneliness; (3) the importance of proximal relationships; and, (4) the importance of networking between proximal relationships, educational institutions and mental health services. 

#### 3.1.1. Theme 1: Internet and Social Media

Participants’ narratives often point to the fact that Internet and social media usage are more and more common among young people nowadays and their use could be responsible for determining suicidal ideation and attempts. They claimed that media usage could be responsible for a weakening of social bonds, alienation from reality and forms of aggression and non-acceptance by peers expressed through cyberbullying:

“*One of the most relevant risk factors is the relationship young people have with the web. Many of them remain prisoners of their computers and mobile phones, drawn into the web where they create virtual superficial relationships thus exiting social reality, isolating*”

(Giuditta)

“*Youngsters, the so called “digital natives” are constantly overconnected on social media, isolating from the external world, withdrawing into themselves and rejecting that affectivity that keeps them earth-bound*”

(Vittoria)

Moreover, participants’ narratives seem to underline the responsibility of media coverage on mental health and suicidal issues and its possible consequences in terms of imitative effects:

“*Moreover, the information and knowledge on this issue that are provided by media, by social, by the news, by movies and by tv series are fundamental because the way they talk about them could lead many people to get to know that phenomenon and to lead many to talk about the difficult times they are facing and about their will to commit the act. At the same time, they could […] push people who are already contemplating it to actually commit the act*”.

(Daniela)

Finally, most participants point to the link between social media usage with phenomena such as bullying and cyberbullying.

“*Among young people it is very common not to be accepted and to be mocked for one’s sexual orientation, one’s physique, one’s culture or one’s religion, and this often brings situations of bullying and cyberbullying, seen by victims as a defeat, a humiliation and they feel so ashamed that the only solution they find is disappearing from society, and therefore killing themselves*.”

(Nicole)

#### 3.1.2. Theme 2: Social Isolation and Loneliness

Participants underlined the role of loneliness and social isolation in determining young people’s choice to take their own lives. In their view, loneliness is in our contemporary society tightly linked with the use of social media. Moreover, social isolation and loneliness have a much stronger impact when they are due to non-acceptance by peers:

“*The fil rouge connecting all youngsters with suicidal thoughts or who died by suicide is the feeling of being alone, isolated, unappreciated and hopeless*.”

(Gioia)

Social media could be held responsible for a certain way of relating to other people that would lead to social isolation and to possibly perceive themselves as lonely:

“*This loneliness is a feature of contemporary society in which we are all very connected, thanks to technology, to social networks, to smartphones but in which we also feel and are very distant from one another. Relationships become more and more superficial, detached and human bonds become “liquid” as Bauman claims. This brings no positive consequences as men are social beings and develop their identity by interacting with other human beings, and they need someone listening to them, emotionally and psychologically supporting them to face life challenges*.”

(Daniela)

Participants claim that given the importance of peers in young people’s lives, non-acceptance by the latter might have detrimental effects on young people making them experience loneliness.

“*Young people then have to face peers, relationships with peers: in particular, with being and feeling accepted by them. […] The exclusion by peers can be a motivation leading young people to kill themselves: an excluded young person might feel different, weird, inadequate, wrong, out of place* […]”

(Emily)

“*One is not beautiful enough, good enough, smart enough. This feeling is also very present within peers, one is always after a sort of perfection to feel like they are worthy, so that others will accept you and validate you. In so doing, one keeps wearing a mask, does not feel like they can show their true personality and this makes them even more insecure, lonely and continuously underestimating themselves. A young person going through such hard times, feeling lonely and unable to be understood by others, that has no self-confidence, that feels like they are not worthy and that when together with other people feels excluded and not accepted is brought to have the extreme thought of suicide*.”

(Carla)

#### 3.1.3. Theme 3: The Importance of Proximal Relationships

Participants’ narratives point to the importance of relationships in young people’s choice to live. More specifically, they underline the importance of friends and family, which are both as valuable and precious, although some of them report friends, as peers, offer the possibility to identify with peers’ life experiences and therefore access a sense of universalism and a feeling to be understood.

They state the importance of their most proximal relationships, meaning family and friends, as main reasons for living:

“*Men are social animals as they tend to aggregate with other individuals and to create societies […]. Living in a nice environment surely leads people to choose life as opposed to its end, offers a reason for their existence, a reason to wake up the next day with the will to live*.”

(Camilla)

Family is considered as one of the most important reasons for living as it can satisfy fundamental human needs such as the need to feel loved, understood and accepted.

“*Feeling loved, surrounded by people with whom one shares an affective bond is for man a protective factor as well as promoting happiness. First of all, there’s family, that gives people their origins, a place where they can feel secure and not judged*.”

(Carla)

“*For people, and particularly for young people, knowing they have on their side someone supporting them, someone “cheering” for them, someone believing in them, someone ready to reach out to them when in need, someone reassuring them to be there despite failures and falls, that can make a difference*.”

(Emily)

Finally, friends are a just as important source of support as family is. Participants nonetheless seem to highlight a qualitative difference in this form of support as young people experience a greater identification with their peers and might therefore feel a greater understanding of their life experiences.

“*For young people it is important to relate to their peers, to confront with their life experiences and to recognize in the other people something similar to them. Relationships with people their age, in fact, allow young people to understand difficulties aren’t just part of their lives and that sharing negative emotions could allow a better awareness and a possible reinterpretation of their thoughts*.”

(Virginia)

“*In case young adults do not have a solid family network or they just do not want to share their problems with family members, a second form of support is that coming from friends. It is likely that young adults would share their problems with friends […] Thanks to their peers, young people can receive recommendations and support from people their age, that could possibly have experienced or be experiencing similar problems*.”

(Margherita)

#### 3.1.4. Theme 4: The Importance of Networking between Proximal Relationships, Educational Institutions and Mental Health Services

Participants point to the importance of the academic environment in determining young people’s choice to live. Moreover, they underline the importance of networking between the most proximal relationships as family and friends with educational institutions and mental health services, to create a support system for young people.

Some participants mention school and university and teachers and professors as important figures in young people’s lives, together with family and friends.

“*Other figures playing a relevant role in young adults’ choice to live are teachers, that young people meet from the beginning of their academic careers. It is in fact important to underline that teachers’ task is not merely padding on knowledge but is also that of accompanying them in their personal growth*.”

(Margherita)

Participants posit schools and universities could be appropriate places where to organize suicide prevention interventions, as captured in the narratives of both Maddalena and Lea.

“*According to me, a fundamental aspect is primary prevention, especially at school. Young adults, in their life path, might get lost and be unable to autonomously choose life, in these cases preventive interventions can help*.”

(Maddalena)

“*School would be a privileged venue where to organize primary prevention interventions, first of all reducing its negative impact, given that it is considered as causing great frustrations to people instead of occasions for growth. In fact, working with teachers to reduce students’ stress levels is a first step to begin transforming schools from a risk to a protective factor […] a place where they could feel comfortable, where they could find and be who they are and where they can find reasons to choose life even if life looks like a dark tunnel with no way out*.”

(Lea)

Finally, participants underline the importance to create a form of network within the social networks of young people, meaning family, peers, high schools or universities and helping professions so as to guarantee the greatest access to services for people experiencing some sort of crisis.

“*The help and collaboration of people surrounding them (relatives, friends, teachers, coaches, …) and of professionals could be a true engine to make the life of that young person take off again […] activating networks that could make the young person feel engaged and appreciated and that are able to catch signals for help they send and to therefore work to support them, implementing appropriate (non/institutional) interventions*.”

(Maddalena)

### 3.2. Project Works

Generally, in their suicide prevention programs, students focused on the reduction of loneliness by strengthening social bonds and networks. Moreover, in almost all of their projects, participants employed creative and art-related ways to work on suicide prevention. Among their projects, university students decided to focus on three different settings for their suicide prevention programs, namely: (1) Suicide Prevention through Community and Networking between Services; (2) Academic Institutions: High schools and Universities; (3) Suicide Prevention through New Technologies.

#### 3.2.1. Suicide Prevention through Community and Networking between Services

Some students decided to prevent suicide by setting their projects within their territory of origins and imagining the potential for community support and networking between the existing services. 

##### Re-Give Me Trust

The first project will be presented in a more extensive way to exemplify how students worked (please see Table 1). 

##### Seven Pounds

The present project work is aimed at 15–21-year-old minors living in residential childcare institutions. The aim of the present study is to present young people different possibilities to face challenging situations. More specifically, the first aim is to strengthen young people’s self-esteem, the second aim is to present young people different problem-solving possibilities when facing hard times and the third aim is to strengthen young people’s social ties and broaden their network of friends. Each meeting will be organized by one residential childcare institution in the territory of the Veneto region in Italy, where all residents from adhering institutions will meet. Students will be first invited to watch the film “Seven Pounds” by Gabriele Muccino in order to start a discussion on suicide and on the value of life. Residents will then be divided into groups and each group will have a chance to change the end of the movie by engaging in a movie-making activity. 

##### Learning to Think

The project is targeted at 18–24-year-old boys and girls with eating disorders. The overall objective of the present project was to prevent suicide risk by both promoting protective factors and reducing risk factors for suicide. More specifically, the project was aimed at: (1) promoting socialization; (2) promoting self-esteem; (3) supporting families; (4) raising awareness on one’s specific eating disorder; (5) raising awareness on eating disorders at the community level. As far as activities are concerned, two types of activities were organized, some for the young girls and boys with eating disorders and some for their parents. Concerning activities for young people, participants thought, for instance, to plan psychodramatic activities to increase self-esteem. Moreover, participants thought they might also create a space in which members of the community could be invited to share their personal experiences with eating disorders and how they managed to recover from them. Simultaneously, participants thought that a similar dialogue with the community might be happening between parents of the young residents with eating disorders and other parents of children who recovered from that same challenging experience.

##### It Depends on Me!

The present project is aimed at 18–25-year-old people struggling with gambling. Overall, the project aims at reducing gambling and thus possibly preventing the onset of suicidal ideation. More specifically, this project is aimed at raising awareness of one’s experience with gambling and thus helping the person recover from this form of addiction by embarking on both personal and group work. During the first phase of the project, guests will gather into small groups and will engage in group psychotherapy activities. In a second phase, participants will participate in self-help groups and psychodramatic activities using a technique called the theatre of the oppressed. 

##### When You Are Down, Drop by!

This project is aimed at 18–25-year-old people suffering from substance abuse. The project is aimed at: (1) creating a safe space alternative to the streets where people with substance abuse can meet; (2) providing psychological help; (3) offering Art-therapy laboratories; (4) creating social opportunities to diminish their perceived loneliness. As regards activities, guests will be invited to join a self-help group and psychotherapy sessions. Moreover, they will be offered to take part in Art-Therapy laboratories such as street art, parkour and video-making. 

#### 3.2.2. Academic Institutions: High Schools and Universities

Some other students focused on suicide prevention set in academic contexts such as high schools or universities and imagined suicide prevention more as a form of psychoeducation aimed at awareness raising.

##### Legames—Suicide Prevention Project through a Responsible Use of Social Networks

The project is targeted at 15–18-year-old high school students and their teachers and parents. The project is aimed at raising awareness on the use of social media and the associated risks in order to educate young students on responsible social media usage. It is targeted at both high school students, and also parents and gatekeepers such as teachers and educators, in order to create a network between family and school to support young people in conscious and safe use of media. First, students will take part in five meetings at school in which they will learn about social media usage, its potential and risks. Then they will be invited to take part in a 10-day summer camp, called “Dix-Connected Week”, in which the use of technology is intended to be prohibited. Parents and teachers will receive education on social media usage through focus groups and psychodramatic activities. 

##### One, No One and One Hundred Thousand

The project is targeted at university students. The overall aims of the present project are to promote group discussions and to broaden students’ support network/system. More specifically, the project is aimed at: (1) creating opportunities to share personal experiences; (2) raising awareness on personality traits; (3) implementing problem-solving strategies; (4) reducing students’ perception of loneliness. Clients will be invited to join group psychotherapy activities in the form of psychodrama and self-help groups. Moreover, at the end of each psychodramatic session, clients will be invited to write their personal journals to further elaborate their experiences. 

##### White Masks

The project is aimed at two targets: Group A are university students within a degree course in which students took their lives; group B are university students. The overall aims of the project are to raise awareness on the issue of suicide and to identify at risk students. More specifically, the project is aimed at: (1) providing students with information that will allow them to recognize warning signs in their peers; (2) offering a space of free debate on one’s emotions, problems and difficulties; (3) directing at risk students to the university psychological support center/service; (4) for group A students only, providing a space in which it is possible to process the loss of a peer. These objectives will be reached through a Death Education course that will employ phototherapy and the organization of a final photographic exhibition where students will share their experience within the project. Moreover, at risk students will be referred to the psychological support service of their university and will receive appropriate individualized support.

#### 3.2.3. Suicide Prevention through New Technologies

Finally, given the numbers of young people employing internet and new technologies, some students decided to do suicide prevention by using new technologies, such as online apps and portals. 

##### Reasons Why

The present project targets 15–25-year-old people. The overall aim of the project is to create a website, called “Reasons why” to prevent loneliness and suicide among Italian youngsters through the creation of social connections. More specifically, the project is aimed at: (1) raising awareness on the issue of suicide; (2) offering psycho-social support and prompting social bonds; (3) promoting positive emotions through the use of arts, creativity and reflection. The website will offer different possibilities, such as a forum with the other users, online counseling support and upload of videos centered on awareness raising on loneliness, bullying and emotional intelligence. Moreover, creative opportunities will be offered, such as photovoice activities and weekly playlists for community users.

##### Taking You by the Arm

The target of this project are 18–24-year-old high school or university students. The overall aim of this project is to create an online application, called “Hand to hand” aimed at suicide prevention. More specifically, the aims of the project are: (1) creating a web portal for chatting with professionals; (2) raising awareness on suicide through dissemination of appropriate scientific material; (3) promoting activities within the community to foster socialization. The application “Hand to hand” will provide users with different opportunities such as online counseling with mental health professionals, updated news about possible events aimed at awareness raising on suicide or socialization and the upload of material on possible useful articles and contacts in case of crisis situations. 

## 4. Discussion

This research sought to broaden the understanding of young people’s concept of suicide and of what they think is the best way to organize suicide prevention among people their age or younger. 

First, this study tried to explore how Social Work university students conceptualize suicidal choices by adolescents and young adults, by enquiring about the reasons which could possibly drive them to such choices or, conversely, prevent them from choosing to die. Students’ narratives point to the importance of Internet and media usage as possible influences on suicide in young people. They first underline that the use of Information and Communication Technologies (ICTs; [41]) is growing, particularly among young people. This is in line with the literature showing that young people who were born after 1995, the so called iGeneration, have grown up in a world immersed in Internet usage and spend about as many as 5 to 6 hours per day engaging in activities such as surfing the net, using social media or texting [42]. The literature also shows that the 18–24-year-old group could be at a higher risk of developing what is known as nomophobia [43]. Nomophobia—stemming from the words “no mobile phone” and “phobia”—is “a disorder of the contemporary digital and virtual society and refers to discomfort, anxiety, nervousness or anguish caused by being out of contact with a mobile phone or computer” [44] (p. 156) and might be linked to the so-called Fear of Missing Out (FoMO), “a pervasive apprehension that others might be having rewarding experiences from which one is absent” [45] (p. 1). 

Furthermore, participants underline a link between ICTs usage and social isolation and loneliness. More specifically, their narratives seem to suggest that the use of new technologies and relative services could increase social isolation in young people which would, in turn, lead to perceived loneliness. In keeping with this claim, a study by Hunt and colleagues [46] shows that decreasing social media usage among university students to 30 minutes per day produces a decrease in both loneliness and depression levels. Nonetheless, most of the studies linking internet and social networking sites usage to loneliness expose an inverse relation, claiming that loneliness might predict nomophobic behaviours [47]. For instance, a meta-analysis by Song and colleagues [48] claimed that instead of the use of Facebook making people lonely, it is lonely people that would tend to use it more often. Overall, loneliness is described by participants as an important drive to suicidal choices in young people. This is in accordance with some recent findings relative to the current COVID-19 pandemic period, pointing to the fact that among the adult population, young adults and particularly girls could suffer the highest increases in loneliness [49,50]. More specifically, the impact of the pandemic would be on their emotional loneliness [49,50], that is a lack in more intimate relationships, as opposed to social loneliness, which refers to the lack of a broader social network [51]. 

Participants’ narratives seem to suggest that more intimate or proximal relationships, like family and friends, are important reasons for living. This result is in accordance with the Reasons for Living Inventory by Marsha Linehan and colleagues [52] and with the ad hoc versions for adolescents and young adults—respectively, the Reasons for Living Inventory of Adolescents (RFL-A; [53]) and the Reasons for Living Inventory for Young Adults (RFL-YA; [54])—in which relationships with family and peers are among the identified factors. In keeping with Putnam’s concept of strong ties [30], participants’ narratives underline that this type of relationship could be able to satisfy young people’s needs for belonging, affiliation and support; connectedness with family and peers shows in fact some potential in suicide prevention programs targeting adolescents, along with school connectedness [55]. Participants highlight the importance of schools and universities in suicide prevention. This is in accordance with the directives by the WHO [56], which considers gatekeeper trainings as useful preventive strategies. Moreover, participants highlight the importance of networking between the most proximal relationships, academic institutions and mental health services, which is in line with literature on suicide prevention programs stating that it is important to strengthen communication between teachers and school counselors or psychologists and to potentiate connections between school and families and school and mental health services available in a relevant territory [6,55]. 

Second, this study sought to explore participants’ ideas regarding suicide prevention potentialities for their peers or for younger people. Some participants highlighted the importance to run suicide prevention programs within their territory, in line with Whitlock and colleague [55]’s definition of connectedness as a “willingness to share with and seek resources from the individuals and communities in which he/she is socially or geographically embedded” (p. 266). For instance, in the project called “Re-give me trust”, participants imagine an exchange and communication between young women who are victims of sexual abuse and old people living in nursery homes as a way to reintroduce these young women into their communities. Thus, young women can engage in activities that give them back a sense of contribution and meaning and allow them to re-construct relationships based on trust, affection and care. Some other participants then point to the importance of suicide prevention programs held at school or in universities and that might be based more on awareness raising on the issue of suicide [57] or on specific potential risk factors for suicide such as problematic media usage [47], which can give rise to phenomena such as nomophobic behaviors or cyber-bullying, which have been shown to have detrimental effects on mental health [58]. Finally, in line with recent attempts to move to web-based suicide prevention interventions [59], some participants point to the importance of taking advantage of the widespread usage of ICTs and using that as a possible venue for suicide prevention.

### Limitations and Future Research

Some limitations were identified in this study. Firstly, the present study is a cross-sectional one which captures the narratives of a limited number of students at a specific moment in time, thus not allowing for longitudinal measures. Secondly, participants in this research are social work university students and therefore offer a specific viewpoint on the present research which cannot be generalized neither to students belonging to other degree programs and levels, nor to the entire population of young people. Future research could explore the narratives of Master’s Degree students or doctoral-level social work students and of social workers. Moreover, it would be interesting to broaden the scope of the participants in order to include students belonging to other healthcare professions, such as psychology students. Finally, another limitation is the great imbalance between the number of female (91.9%) and male (8.1%) participants in the present study, which might reflect the choice of college programs by students.

## 5. Conclusions

The present research aimed at exploring Social Work university students’ understanding of the reasons driving young people to or preventing them from suiciding. Moreover, this research also wanted to explore how university students picture possible suicide prevention interventions targeting their peers and adolescents. On the one hand, as far as reasons for dying are concerned, young people’s narratives seem to suggest the important role of loneliness, which seems to be intertwined with the use and, maybe, abuse of internet and social media. On the other hand, participants seem to suggest the important role played by their most proximal relationships, such as their families and friends, in shaping their choice to welcome life. Moreover, participants acknowledge the role of schools and universities in suicide prevention and the importance to create networks between young people’s most proximal relationships, academic institutions and mental health services in order to support young people in times of crisis. Furthermore, they seem to point to the complexity of the relationship with ICTs, which are identified as a potential resource for suicide prevention interventions, given their spread among young people. Since this research was carried out before the outbreak of the Covid-19 pandemic, future research might explore if and how the pandemic and the consequent changes it brought about might have influenced college students’ narratives in regard of their reasons for living and for dying and on potential resources for suicide prevention interventions. To conclude, Death Education in the form of education on suicide prevention can offer young people a space in which to ponder on theirs and their peers’ reasons for living and dying and to create their personal narrative on the meaning life has for them [15,16]. 

## Figures and Tables

**Table 1:** Example of the project titled “Re-give me trust”.

Target	18–25-Year-Old Women Victims of Sexual Abuse
**Setting**	Headquarters of the Re-give me trust association for victims of sexual abuse in northern Italy
**Objectives**	The aims of the present project were to empower and increase self-esteem, restore trustworthy relationships with other people and rehabilitate young women within their communities. In order to achieve such goals, students hypothesized a possible cooperation between different actors involved in health services such as family counseling centers, mental health centers and nursery homes
**Équipe and Services**	Social workers; psychologists; psychiatrists; educators; voluntary workers; Mental health center; family counseling center; nursery homes
**Examples of activities**	- meeting with social workers - psychological and medical assessment - group psychotherapy - participation in self-help groups, in order to reintroduce women within their communities - voluntary work activities, for instance in nursery homes, where young women could have an opportunity to interact with guests and restore positive relationships based on affection, care and trust

## Data Availability

The data presented in this study are available on request from the corresponding author. The data are not publicly available due to privacy protection issues.
